# Single-Cell Cortical Transcriptomics Reveals Common and Distinct Changes in Cell-Cell Communication in Alzheimer’s and Parkinson’s Disease

**DOI:** 10.1007/s12035-024-04419-7

**Published:** 2024-08-15

**Authors:** Sophie Le Bars, Enrico Glaab

**Affiliations:** Biomedical Data Science Group, Luxembourg Centre for Systems Biomedicine (LCSB), University of Luxembourg, Esch-sur-Alzette, Luxembourg

**Keywords:** Alzheimer’s disease, Parkinson’s disease, Cross-disease comparison, Single-cell analysis, Cell-cell communication, RNA-sequencing, Pathway analysis, Network analysis

## Abstract

Alzheimer's disease (AD) and Parkinson's disease (PD) cause significant neuronal loss and severely impair daily living. Despite different clinical manifestations, these disorders share common pathological molecular hallmarks, including mitochondrial dysfunction and synaptic degeneration. A detailed comparison of molecular changes at single-cell resolution in the cortex, as one of the main brain regions affected in both disorders, may reveal common susceptibility factors and disease mechanisms. We performed single-cell transcriptomic analyses of *post-mortem* cortical tissue from AD and PD subjects and controls to identify common and distinct disease-associated changes in individual genes, cellular pathways, molecular networks, and cell-cell communication events, and to investigate common mechanisms. The results revealed significant disease-specific, shared, and opposing gene expression changes, including cell type-specific signatures for both diseases. Hypoxia signaling and lipid metabolism emerged as significantly modulated cellular processes in both AD and PD, with contrasting expression alterations between the two diseases. Furthermore, both pathway and cell-cell communication analyses highlighted shared significant alterations involving the JAK-STAT signaling pathway, which has been implicated in the inflammatory response in several neurodegenerative disorders. Overall, the analyses revealed common and distinct alterations in gene signatures, pathway activities, and gene regulatory subnetworks in AD and PD. The results provide insights into coordinated changes in pathway activity and cell-cell communication that may guide future diagnostics and therapeutics.

**Supplementary Information:**

The online version contains supplementary material available at 10.1007/s12035-024-04419-7.

## Introduction

Alzheimer's Disease (AD) and Parkinson's Disease (PD) are the two most common neurodegenerative disorders, and their prevalence continues to increase in the aging population [1]. AD is characterized by the aggregation of the protein tau and the accumulation of amyloid beta (A*β*) plaques, leading to progressive cognitive impairment and eventually dementia [2]. By contrast, PD is characterized by a pathological accumulation of the protein *α*-synuclein and dopamine depletion in vulnerable brain regions, associated with the cardinal motor symptoms of bradykinesia, rigidity, and resting tremor. While AD has a higher age-adjusted incidence rate in women compared to men [3], PD predominantly affects men [4].

Despite their distinct molecular and clinical profiles, AD and PD share many similarities. Central to these similarities are biological processes related to oxidative stress, neuroinflammation, iron homeostasis, and neuronal loss [5, 6].

In addition, the disorders manifest similar comorbidities, including memory changes, sleep disturbances, communication difficulties, behavioral changes, and cognitive decline [7, 8]. Investigating the convergence and divergence of cellular pathways involved in these disorders may help to better understand the underlying biological mechanisms and advance therapeutic development. Emerging evidence highlights the presence of shared molecular susceptibility factors and pathways, providing the basis for a mechanistic interpretation of the molecular commonalities between AD and PD. This exploration promises to provide new insights into potential common therapeutic targets and avenues for intervention.

Many previous studies have already conducted comparative analyses of neurodegenerative disorders, including AD and PD, mostly focusing on existing therapeutic modalities [1] or exploring their associations with the aging process [9]. These studies have revealed disease commonalities that contribute to our understanding of neurodegeneration. However, cross-disease comparisons of molecular data have mostly relied on bulk level measurements from different tissues and body fluids, precluding an in-depth analysis of single-cell differences.

To date, a comprehensive comparison of molecular profiles in PD and AD at single-cell resolution in commonly affected brain regions is still lacking. To help fill this gap, we collected single-cell RNA-seq (scRNA-seq) data from AD and PD subjects and controls, focusing on the cortex as a key brain region implicated in both diseases. Using these data, our study characterizes the common and distinctive features between AD, PD, and controls in single cells at multiple levels, from individual genes to pathways and subnetworks. By using a single-cell based multi-level approach, we aim to provide both coarse and fine scale comparisons, capturing detailed changes in individual genes and overarching changes in pathways/subnetworks to gain a more comprehensive understanding of the underlying processes in each cell type.

Overall, our comparative single-cell transcriptome analysis revealed unique and shared gene signatures and pathway changes between AD and PD. Focusing on five major cell types common to both datasets (astrocytes, oligodendrocytes, microglial cells, excitatory neurons, and inhibitory neurons), we identified significant contrasting alterations, particularly in pathways associated with synaptic dysfunction and lipid metabolism. In addition, hypoxia-related and inflammation-related pathways, such as the JAK-STAT signaling pathway, were highlighted as significant in multiple analyses. Furthermore, network analyses revealed key regulatory genes whose modulation has the potential to reverse downstream AD- and PD-associated expression changes in the networks. These include the transcription factor *CREB1*, which regulates neuronal plasticity via the CREB signaling pathway, and central regulatory genes involved in stress response pathways such as *JUNB, FOS,* and *HIF1A.*

In summary, our study provides insights into the molecular commonalities and differences between AD and PD at single-cell resolution across multiple levels of biological organization, encompassing the hierarchy of genes, pathways, and molecular networks. The findings may facilitate the development of improved disease-specific diagnostics, while also revealing shared susceptibility factors and pathways, including pharmaceutically tractable target proteins with favorable druggability characteristics.

## Methods

### Alzheimer’s and Parkinson’s Disease Single-Cell Datasets

We used two distinct scRNA-seq datasets, one from an AD cohort and one from a PD cohort.

The AD dataset was obtained from *post-mortem* dorsolateral prefrontal cortex (DLPFC) samples from 71 AD subjects and 9 healthy controls (HC) [10]. Nuclei were isolated using a previously described standard procedure from Allen Institute [11]. The nuclei were then concentrated by centrifugation and sequenced on a 10X Chromium platform (10X Genomics, see [10] for the detailed experimental procedures). We obtained the pre-processed and annotated single-cell data from https://registry.opendata.aws/allen-sea-ad-atlas.

The PD dataset was derived from *post-mortem* prefrontal cortex samples from 6 idiopathic PD subjects and 6 matched HC [12] (GEO: GSE202210). Brain nuclei were isolated by sucrose gradient ultracentrifugation, and single nucleus libraries were constructed using the 10X Chromium system and sequenced on an Illumina NovaSeq 6000 system, yielding approximately 80,000 nuclei (see [12] for experimental details).

**Fig. 1:**
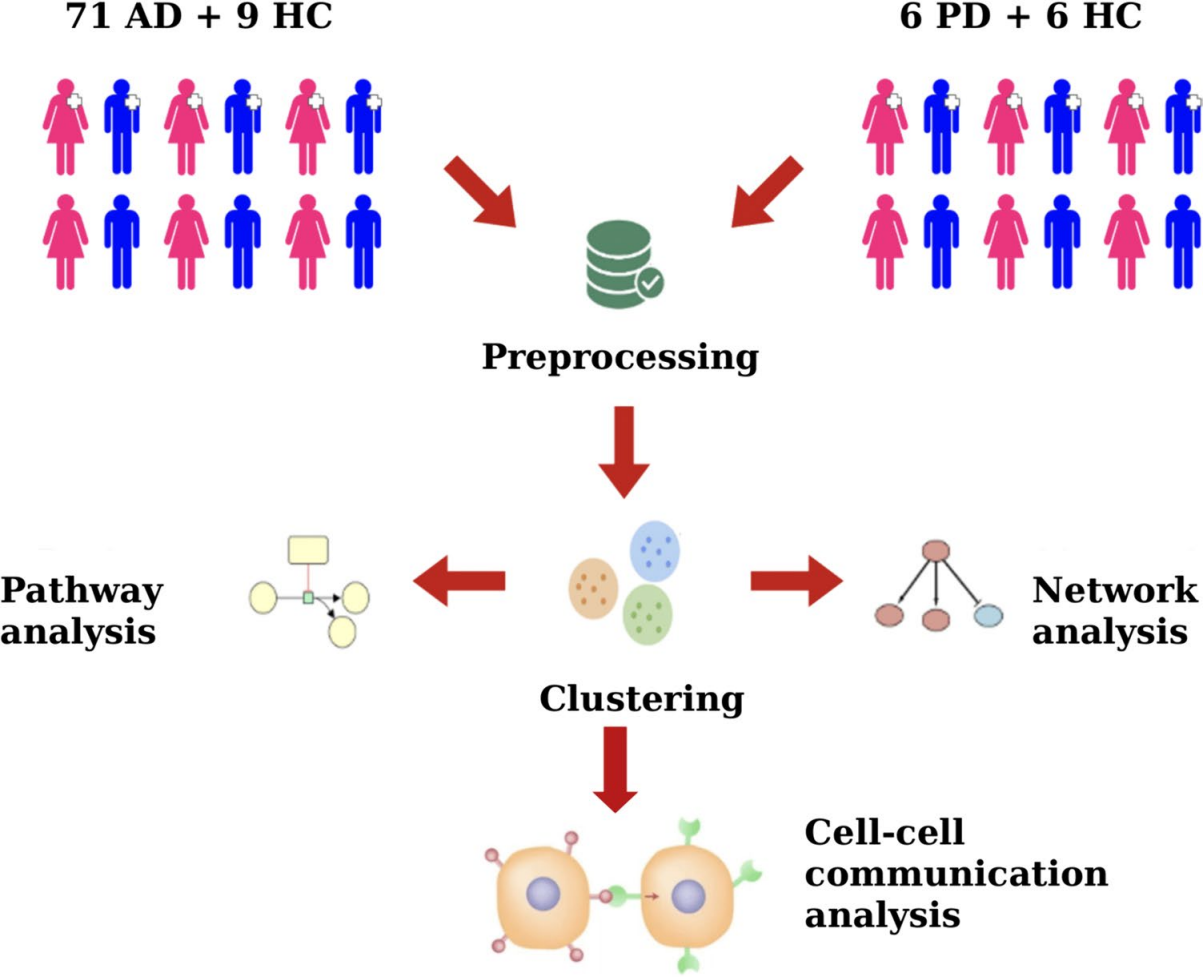
Workflow for the comparative analysis of scRNA-seq data in Alzheimer’s (AD) and Parkinson’s Disease (PD). For the AD dataset, cluster annotations were derived from the original source data by Gabitto et al. (2023) and validated using the CellMarker database; for the PD dataset, clustering and cell type annotation were performed as described in the Methods

An overview of the main characteristics of the two datasets, including information on the represented conditions, Braak progression stages, ages, sexes, and *post-mortem* intervals is provided in Table 1. The overall workflow for the pre-processing and analysis of scRNA-seq data in AD and PD is illustrated in Fig. 1.

**Table 1:** Overview of the AD and PD single-cell datasets (Cohort = disease condition studied in the cohort, Groups = number of samples per condition (HC = healthy controls), Sex = number of samples per biological sex (F = female, M = male), Age = age in years, Braak = Braak progression stage ranging from 1 (low) to 6 (high), PMI = *post-mortem* interval in hours)

Cohort	Groups	Donors (n)	Sex M / F (%)	Age (mean ± sd)	Braak (mean ± sd)	PMI (mean ± sd)	Total number of nuclei (n)
AD	AD	71	40% M / 60% F	88.6 ± 8.0	4.6 ± 1.13	6.9 ± 2.15	1300229
	HC	9	52% M / 48% F	89.3 ± 5.74	3.0 ± 1.68	6.3 ± 2.15	
PD	PD	6	50% M / 50% F	81.8 ± 8.32	1.5 ± 0.83	7.6 ± 2.80	77384
	HC	6	50% M / 50% F	71.5 ± 6.32	1.2 ± 0.75	10.0 ± 4.90

### Pre-Processing, Quality Control and Filtering

Single-cell data pre-processing was performed using the R software package Seurat for the PD cohort (version 5, RRID:SCR 016341) [13]. Following standard Seurat guidelines, we applied dedicated quality control (QC) checks to remove outlier cells (gene counts below 200 or above 10000) and cells with mitochondrial contamination (percentage of cells with *>* 5% mitochondrial counts), as well as empty droplets or doublets (droplets containing two cells; see also the Data Availability section for the source code).

As a result, we obtained 35555 PD cells and 29865 HC cells for the PD dataset. Finally, we applied normalization, scaling and variance stabilization using the function *scTransform* in the Seurat package, using regularized negative binomial regression. This initial pre-processing also involved an unsupervised feature selection, reducing the number of features to the top 3000 most variable genes. All data pre-processing and analysis steps were performed in the R programming language (version 4.4.0, RRID:SCR 001905) [14]. All computations were performed using an RStudio Server with 1 TB of memory to handle the large cell counts in the data.

For the AD cohort, we classified participants as AD or HC according to the ADNC score (0 for HC, greater than 0 for AD), as used in Gabitto et al. (2023). We included all cells from the 80 donors which met the QC criteria defined in Gabitto et al. (2023) and we verified that the mitochondrial count for these cells was less than 5%.

### Clustering and Cell Type Annotation

To identify and annotate the cell populations in the PD single-cell dataset, we first applied a dimension reduction using Principal Component analysis (PCA) and the Uniform Manifold Approximation and Projection (UMAP) for data visualization. Specifically, the first 17 principal components (PCs) were selected using the Elbow method in the Seurat package for further downstream analysis. Next, a Shared Nearest Neighbor (SNN) graph was constructed using the selected PCs and the *FindNeighbors* function in Seurat. Cell type clusters were then identified in the SNN graph using the *FindClusters* function in Seurat and the Louvain algorithm [15]. To determine the optimal number of clusters, we computed the Silhouette width cluster validity score [16], using the R packages cluster [17] (version 2.1.6, RRID:SCR 013505) and clustree (version 0.5.0, RRID:SCR 016293) [18]. The Silhouette width assesses both cluster separation and compactness, while the clustree package was used to analyze and visualize cluster stability and distribution characteristics. Finally, the resulting cell type clusters were annotated using the ScType software [19] and markers from the CellMarker database. For further confirmation, the identified cell types were cross-referenced against the previous reference publication [12].

### Gene-Level Analysis of Disease-Associated Changes

We performed gene-level differential analyses for the main cell types thought to play important roles in AD and PD, including excitatory and inhibitory neurons, oligodendrocytes, microglial cells, and astrocytes. For this purpose, we used the non-parametric Wilcoxon rank sum statistic, as implemented in the *FindMarkers* function of the Seurat package, to identify differentially expressed genes (DEGs) between patients and controls in the respective cohorts (PD vs. HC / AD vs. HC). All p-value significance scores were adjusted for multiple hypothesis testing using the Benjamini-Hochberg procedure. If the resulting false discovery rate (FDR) for a gene was less than 0.05 and a minimum effect size was observed (absolute log fold change ≥ 0.10), the corresponding gene was considered as significantly differentially expressed. Using this procedure, we determined significant DEGs for both cohorts and grouped them into three main categories:

- *Shared DEGs*: Genes with shared significant differential expression and the same direction of the change in both diseases (FDR *<* 0.05, min. absolute log fold change *>* 0.10 for both diseases and identical signs of the log fold change)
- *Disease-specific DEGs*: Genes that are significantly differentially expressed in one of the diseases (FDR *<* 0.05, min. absolute log fold change *>* 0.10) and not close to significance in the other disease (nominal p-value *>* 0.5) - this conservative threshold for non-significance was chosen to ensure that a disease-specific significance is not assigned due to stochastic variation of FDR values around the standard significance threshold of 0.05)
- *Contrasting DEGs*: Genes with significant differential expression in both diseases, but changes in different directions (FDR *<* 0.05 and min. absolute log fold change *>* 0.10 for both diseases and different signs of the log fold change)

The relative proportions of these three categories of DEGs across the main cell types of interest were visualized using Venn diagrams (R package nVennR, version 0.2.3).

### Pathway Analysis

To characterize disease-associated changes at the scale of cellular pathway and process activities, we evaluated the overrepresentation of DEGs in biological processes (BP) and molecular functions (MF) from the Gene Ontology (GO) database and in pathways from the KEGG database (KEGG). The common pathway alterations between AD and PD were investigated by performing the analysis separately for two categories of identified DEGs: shared and contrasting DEGs (see definition in the section on "Gene-level analysis of disease-associated changes"). Overrepresentation analysis was performed using the *enrichGO* and *enrichKEGG* functions from the R package *clusterprofiler* (version 4.2.2, RRID:SCR 016884) [20] and human gene annotations from the R package *org.Hs.eg.db* (version 3.10.0) and the KEGG database. Results were considered significant if the adjusted p-value was less than 0.05 and the number of DEGs in the enriched pathway was above 5. All analyses were carried out for all main cells considered also in the gene-level analysis of disease-associated changes. Finally, we examined the directionality of the changes in the DEGs mapped to the GO and KEGG gene sets to assess global trends of increasing or decreasing gene expression in the two diseases for each gene set analyzed.

### Subnetwork Analysis

Next, we conducted gene regulatory network perturbation analysis as previously described [21], to identify key regulatory genes/proteins (here called perturbagens), whose pharmacological modulation has the potential to reverse downstream pathologic changes in the gene regulatory networks (GRNs). Because this method requires input networks of sufficient size and connectivity to identify essential circuits for maintaining network stability, it was only applicable to the larger networks obtained in the GRN construction analysis, and we therefore used it specifically to identify perturbagens for the astrocyte, oligodendrocyte, excitatory neuron, and microglial cell types. For all GRNs, we also used an alternative approach to identify key regulatory genes, by performing network topological analysis to determine genes with a high network centrality, as measured by the degree score. The corresponding analysis and visualizations were implemented using the Cytoscape software [22].

### Cell-Cell Communication Analysis

To improve our understanding of cell-cell communication changes in the studied neurodegenerative diseases, we have adopted a new statistical approach to identify and score changes in cell-cell communication, implemented in the R package scSeqComm (version 1.0.0) [23]. Using this software, we integrated the annotated single-cell data with transcriptional regulatory network data and receptor-transcription factor association data using information from the databases KEGG, TRRUST v2, HTRIdb, GO and RegNetwork, and a Ligand-Receptor interaction collection from NicheNet [24]. This allowed us to assess both intra- and inter-cellular communication in our datasets, facilitating the understanding of cellular pathways affected by altered communication events in AD and PD. We focused specifically on astrocytes and microglial cells, which play central roles in neurodegenerative diseases and displayed significant pathway alterations in our data. Cell-cell communication is quantified through inter- and intracellular signaling scores, named *S*_*inter*_ and *S*_*intra*_*,* respectively, which range from 0 to 1, where higher values indicate stronger evidence for communication. We chose a minimum threshold of S_*inter*_ and S_*intra*_ signaling scores of 0.8 for both gene expression datasets to consider only the most specific ligand-receptor pairs. In addition, we focus on discussing the most significant findings, particularly on the cellular processes in the GO database with a p-value below 0.05 that are shared across both diseases in the cell types displaying large numbers of specific LR pairs, i.e. microglial cells and astrocytes.

## Results and Discussion

### Cell Type Clustering and Annotation

**Fig. 2:**
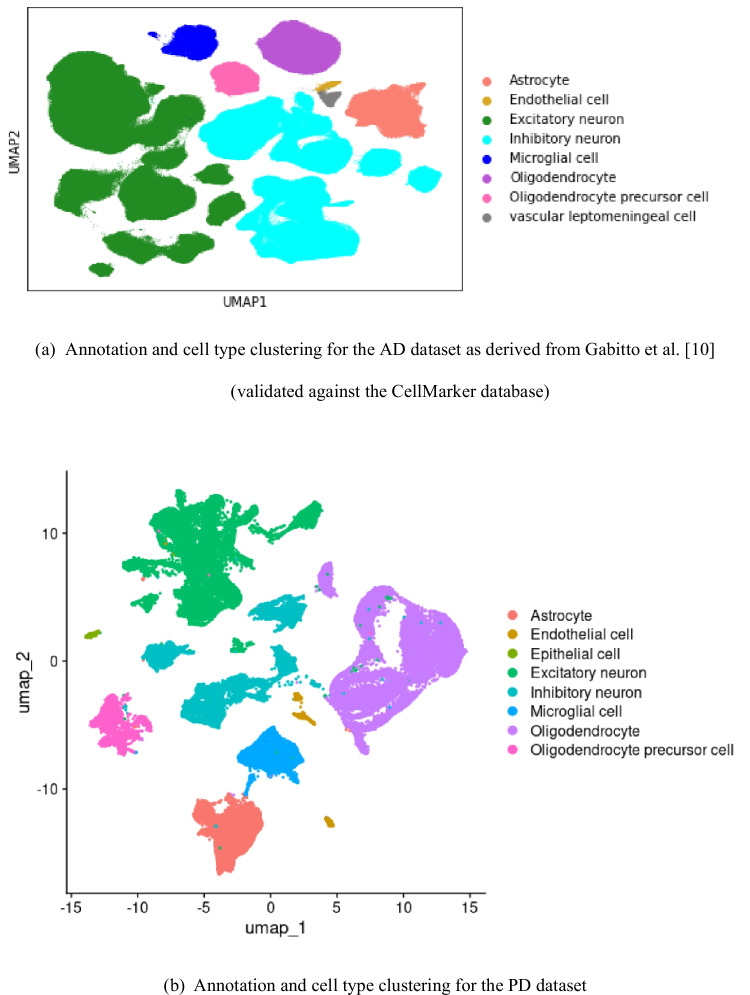
Two-dimensional cluster visualization of the scRNA-seq datasets for (**a**) AD, showing the cell type assignments from the original SEA-AD study (validated against the CellMarker database), and for (**b**) PD, generated using the UMAP dimension reduction approach and highlighting the added cell type annotations (see Methods)

The clustering results for the pre-processed scRNA-seq datasets for AD and PD and the annotations for the corresponding cell type clusters are shown in Fig. 2. For the PD dataset, we confirmed the obtained annotations by visualizing cell type-specific marker genes derived from the CellMarker database and manually inspecting the literature on the marker genes for all cell type clusters visible in the UMAP representation (see Supplementary Fig. 1). These markers exhibit significant overexpression in the respective clusters, as confirmed by assessing differential gene expression between the clusters. For the AD dataset, we validated the annotations from the original source publication [10] by performing a differential expression analysis comparing each cell type cluster against all others and comparing the resulting cell cluster-specific DEGs against known marker genes in the CellMarker database.

In the PD dataset, 10 clusters were identified as the optimal number of clusters using the Silhouette width evaluation. High-confidence cell type annotations were obtained for each of these clusters when applying the ScType algorithmic annotation approach using data from the CellMarker database. Overall, 8 main distinct cell types were detected for both the AD and PD datasets (see Fig. 2), including cell type annotations mapping to multiple point clusters, indicating the potential to investigate finer subdivisions of cell populations in future follow-up studies. To focus on the most robust data patterns with statistical support from large cell counts, we merged cell types for smaller clusters with overlapping differential gene markers and similar annotations for both the AD and PD datasets. Specifically, the clusters labeled as different subcategories of excitatory or inhibitory neurons were merged under the generic terms "Excitatory neuron" and "Inhibitory neuron". Overall, we identified several relevant cell types in the data from both cohorts that have been reported previously to display molecular alterations in neurodegenerative diseases, including neurons, astrocytes, oligodendrocytes, and microglial cells [25], among others. Therefore, the statistical analysis and cellular pathway and network analysis described below focus on these common cell types with confirmed disease relevance and enough cells for robust analysis.

### Gene-Level Comparative Analysis

**Fig. 3:**
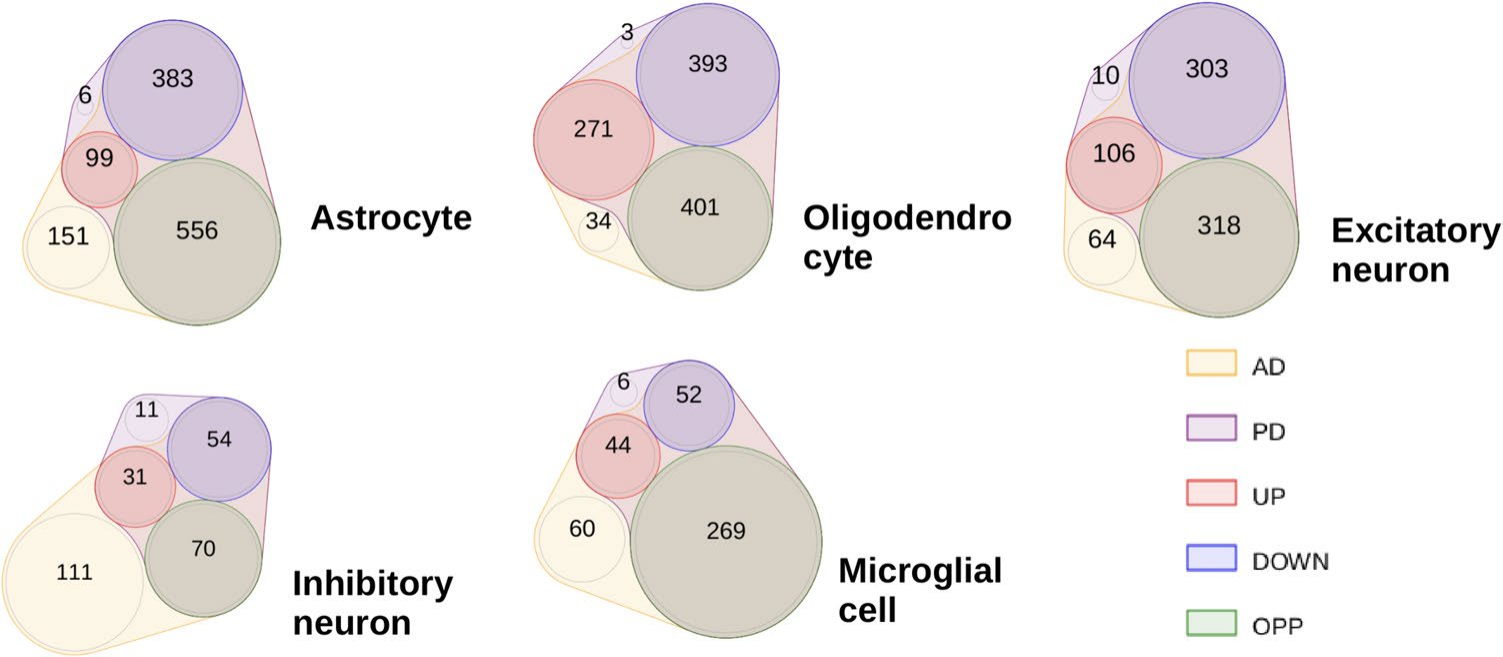
Venn diagram visualizing the intersections between differentially expressed genes (DEGs) in Parkinson’s disease (PD) and Alzheimer’s disease (AD): Disease-specific DEGs are shown in yellow (AD) and purple (PD), shared DEGs with joint increased expression in red, joint decreased expression in blue, and shared significance but opposite direction of the change in green. Intersections are shown for the combination for the five specific cell types of interest (excitatory and inhibitory neurons, astrocytes, oligodendrocytes, and microglial cells)

The statistical analysis of gene-level differential expression between AD vs. HC and PD vs. HC revealed numerous significantly differentially expressed genes (DEGs) for both diseases. The DEGs are categorized into three groups as defined in the Methods section: Shared DEGs (highlighted in red for increased expression and blue for decreased expression), disease-specific DEGs (shown in yellow for AD and purple for PD) and contrasting DEGs (highlighted in green). The numbers and overlaps of DEGs are indicated for the five main cell types of interest: excitatory and inhibitory neurons, astrocytes, oligodendrocytes, and microglia. Several significant DEGs were identified for each category (see Fig. 3). In general, more AD-specific than PD-specific DEGs were detected, which may be explained by the disparity in the cell counts between the two datasets (≈ 1.3M nuclei for AD vs. 80k nuclei for PD), although other factors may also contribute to the overall difference in DEG counts, such as differences between the disease stages covered and disease-specific variations in the extent of cortical molecular changes. Additionally, technical differences such as different methods for nuclei isolation between the AD and PD datasets (see Methods) can impact the yield and quality of isolated nuclei. Despite the potential noise introduced by these methodological differences, their impact on the variability in the integrity and yield of isolated nuclei is considered manageable, allowing for meaningful downstream analyses and comparisons of scRNA-seq results.

As a general observation, the analysis reveals numerous overlapping DEGs between the diseases, with an intersection set size larger than expected by chance according to Fisher’s exact test (p < 0.05 for all cell type-specific analyses). While most of these shared significant DEGs display alterations in the same direction, interestingly, a subset shows changes in opposite directions (illustrated in green). These contrasting DEGs (see definition in the Methods section on "Gene-level analysis of disease-associated changes"), along with the identified disease-specific DEGs, may serve as candidate markers to discriminate between the different neurodegenerative conditions, and provide starting points to investigate disease-specific molecular and cellular mechanisms (see also the following pathway and network analysis). To highlight the main shared and distinct DEGs identified, Table 2 lists the top 5 most significant DEGs for each category, indicating the direction of the change (blue for decreased expression and red for increased expression; log fold changes, p-values and detailed annotations for these genes are provided in the Supplementary Tab. 1). For the contrasting DEGs, arrows indicate whether their expression increases (↗) or decreases (↘) in PD (left arrow) or AD (right arrow). All DEGs for all shared cell types are reported by category, with detailed statistics on the accompanying GitLab website (https://gitlab.com/uniluxembourg/lcsb/biomedical-data-science/bds/comparison_ad_pd_single-cell.git).

**Table 2:**
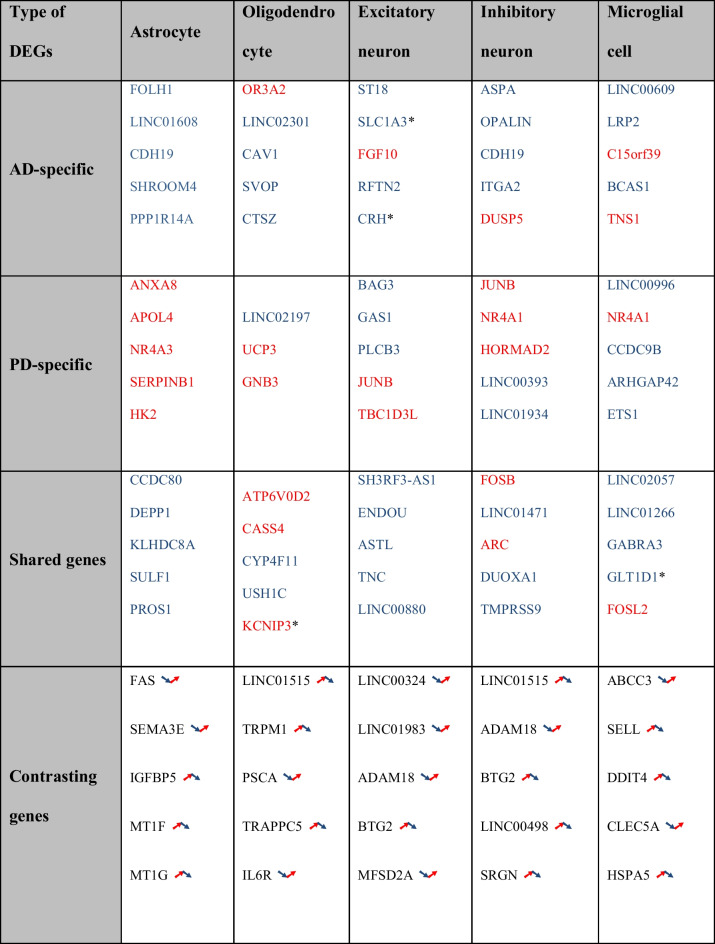
Overview of the most significant differentially expressed genes (DEGs), categorized by disease-specificity (AD-specific, PD-specific, shared DEGs, and contrasting DEGs that share only significance but not the direction of the change) and the cell types considered (excitatory and inhibitory neurons, astrocytes, oligodendrocytes, and microglia). DEGs are highlighted in blue for decreased expression and in red for increased expression. For the contrasting DEGs with opposite directionality in PD and AD, arrows indicate whether their expression increases (↗) or decreases (↘) in PD (left arrow) and in AD (right arrow). An asterisk indicates that the DEG is annotated as a marker gene for the cell type in question in the CellMarker database, and its differential expression may therefore reflect a different representation of the cell type subpopulation between conditions

#### Gene Function Analysis

Among the most significant DEGs listed in Table 2, we note several key functional groups:

1. **Cell Adhesion and Cell-Cell Communication:** Genes such as *CDH19, CAV1, ITGA2, BCAS1, CCDC9B, ARHGAP42, CCDC80*, and *SELL* play important roles in cell adhesion and cell-cell communication. Disruptions in these processes can lead to impaired neuronal connections and signaling, which are central to the progression of both AD and PD [26–28].
2. **Transcriptional Regulation:** This group includes genes such as *JUNB, NR4A1, ETS1, FOSB, and FOSL2*. Abnormal transcriptional regulation can affect numerous cellular functions and is a common feature in the pathology of neurodegenerative diseases, contributing to the dysregulation of gene expression [29–32].
3. **Neurotransmitter Transport and Signaling:** Key genes include *SLC1A3, SVOP, GABRA3,* and *GLT1D1*. Dysfunctions in neurotransmitter transport and signaling are hallmark features of both AD and PD, resulting in impaired synaptic communication and neuronal death [33].
4. **Cellular Stress Response and Apoptosis:** Genes such as *PPP1R14A, SERPINB1, BAG3, TBC1D3L, JUNB, DDIT4,* and *HSPA5* are important for managing cellular stress and apoptosis. Increased cellular stress and improper apoptosis contribute to neuronal loss and the progression of neurodegeneration [6, 34].
5. **Developmental Processes and Growth:** This group includes *FGF10, CRH, SEMA3E,* and *IGFBP5*. Aberrations in developmental processes and growth factors can impact neuronal development and regeneration, which are affected in AD and PD [35–38].
6. **Immune Response and Inflammation:** Genes such as *CTSZ, GAS1,* and *IL6R* are involved in the immune response and inflammation. Chronic inflammation is considered a significant factor in the pathogenesis of both diseases, driving further neuronal damage [39].
7. **Vesicle Trafficking and Intracellular Transport:** *ST18, LRP2, PROS1,* and *TMPRSS9* are involved in vesicle trafficking and intracellular transport. Disruptions of these processes can lead to impaired protein and organelle transport, contributing to the cellular dysfunction observed in neurodegenerative diseases [40].
8. **Cellular Signaling and Regulation of Cellular Processes:** Genes such as *RFTN2, DUSP5, GNB3, PLCB3, CASS4,* and *SH3RF3-AS1* are involved in cellular signaling and regulation. Alterations in these pathways can disrupt normal cell functions and contribute to the pathophysiology of AD and PD [41, 42].
9. **Regulation of Neuroplasticity / Fos Family Genes**: *FOSB* and *FOSL2*, members of the Fos family, are involved in transcriptional regulation linked to neuroplasticity. These genes are associated with cognitive dysfunction, as they influence processes such as memory formation and synaptic plasticity [43].
10. **Maintenance of Neuronal Excitability / Excitotoxicity-Related Genes**: The genes *SLC1A3* and *GABRA3* are both associated with the regulation and maintenance of neuronal excitability. *SLC1A3* gene encodes a glutamate transporter, which is important for regulating glutamate levels in synaptic regions. Its dysfunction can lead to excitotoxicity and has been reported to contribute to neuronal damage in AD and PD [44]. *GABRA3* encodes a subunit of the GABA-A receptor, playing important roles in GABAergic signaling and in the general maintenance of neuronal excitability and prevention of excitotoxicity. Alterations in GABAergic signaling are implicated in various brain diseases, including PD, where disrupted inhibitory signaling can contribute to motor and cognitive symptoms [45].

These diverse groups of the top significant DEGs illustrate the complex and multifaceted nature of the molecular changes involved in AD and PD. Overall, they highlight relevant cellular processes, regulatory factors, and mechanisms that have previously been implicated in the pathogenesis of AD, PD, or other neurodegenerative disorders.

#### Neuroprotective and Neurotrophic Genes

In addition to potential shared disease susceptibility genes, we also investigated the occurrence of genes with neurotrophic or neuroprotective functions among the DEGs. A corresponding curated collection of neurotrophic/neuroprotective genes associated with *in vitro* and *in vivo* evidence from biomedical literature has been assembled in the public database NeuroProDB (neuroprodb.net). The identified neurotrophic/protective DEGs are listed in Supplementary Table 2.

A protective DEG of interest with a contrasting change between AD and PD in astrocytes and inhibitory neurons is *MT3* (metallothionein 3), which encodes a metal-binding protein induced under hypoxic conditions. *MT3* has been reported to protect against oxidative stress by contributing to the removal of reactive oxygen species [46] and its diverging expression alterations in AD (under-expressed in both cell types) and PD (over-expressed in both cell types) indicate that it may be involved in the two diseases through different mechanisms. However, according to the CellMarker database, *MT3* is also a marker gene for astrocytes and astrocyte sub-populations [47]. Thus, diverging alterations in *MT3* expression may also reflect differential representations of subpopulations of cells and need to be interpreted with caution.

An example of a shared DEG with neuroprotective functions detected across multiple cell types is *VEGFA* (Vascular Endothelial Growth Factor A), a growth factor involved in the regulation of vascularization and angiogenesis [48]. *VEGFA* shows significantly increased expression in both neurodegenerative diseases in astrocytes, inhibitory neurons, and oligodendrocytes and may therefore represent a protective mechanism with broad relevance across different degenerative disorders. *VEGFA's* neuroprotective role is underscored by its involvement in promoting angiogenesis and mitigating neuronal damage in the context of AD and PD, and previous studies have suggested potential therapeutic applications aimed at enhancing its expression or function [49, 50].

### Comparative Pathway Analysis

Pathway enrichment analysis was performed for the main cell types of interest (see Methods), and comprehensive ranking tables for all pathways and DEG categories are provided on a dedicated GitLab webpage (https://gitlab.com/uniluxembourg/lcsb/biomedical-data-science/bds/comparison_ad_pd_single-cell.git). Since a detailed coverage of the pathway analysis results for all cell types and all categories of DEGs would extend beyond the scope of this study, we present here as a representative example the enriched pathways for shared and contrasting DEGs for the five main cell types of interest, which displayed the largest numbers of DEGs. For each of these cell types, an overview of the top 3 most significant molecular functions and biological processes in the Gene Ontology database, and pathways in the KEGG database enriched for shared and contrasting DEGs is provided in Table 3.

**Table 3:**
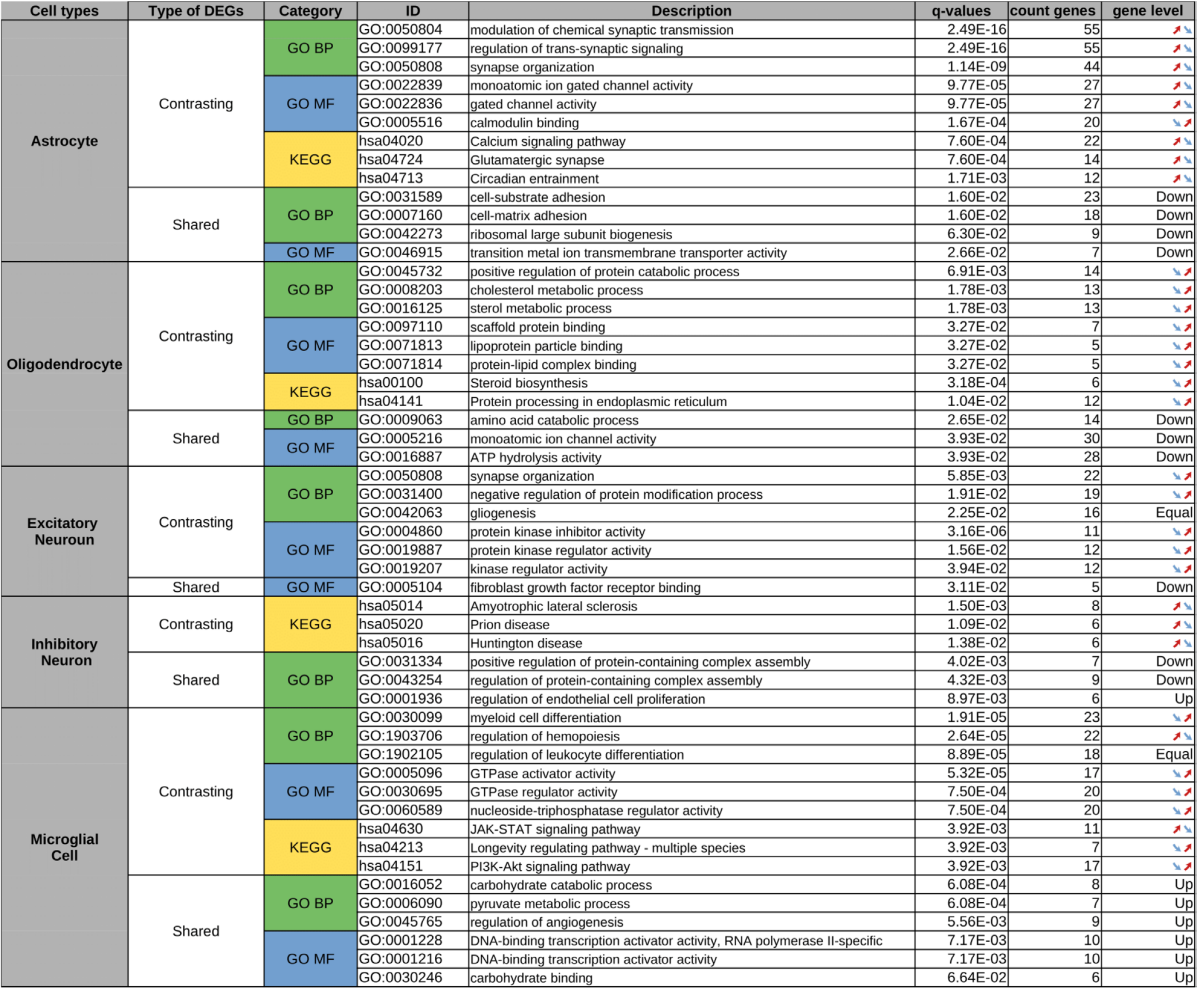
Overview of the top 3 most significant biological processes (BP) and molecular functions (MF) from the Gene Ontology database and pathways from the KEGG database with an overrepresentation of DEGs. Two categories of common significant DEGs between AD and PD were considered as input, those with shared (identical sign of the log fold change) and those contrasting expression changes (opposite sign of the log fold change). The labels "Up" and "Down" in the last column indicate the direction of the change when the majority of shared expression changes between AD and PD within a pathway/process display the same direction across different pathway members. The label "Equal" in the last column indicates that the same number of DEGs with increased or decreased expression was present in the corresponding pathway/process. For the contrasting DEGs with opposite directionality between PD and AD, arrows indicate whether their expression increases (↗) or decreases (↘) in PD (left arrow) and in AD (right arrow)

These results indicate that several cellular processes and pathways display significant alterations in both AD and PD, including both cell type-specific changes and changes shared across multiple relevant cell types. Six categories of significantly altered processes stand out in terms of prior evidence for their relevance in the context of neurodegenerative disorders:

#### Synaptic Dysfunction

We observe multiple pathways and molecular functions related to synaptic processes that display a significant overrepresentation in the contrasting DEGs in both astrocytes and excitatory neurons. The dysregulation of synapse organization is a well-documented hallmark of both PD and AD [51]. Notably, the biological process "Synapse organization" (GO:0050808) is enriched in contrasting DEGs for both astrocytes and excitatory neurons, and the pathway "Glutamatergic synapse" (KEGG, hsa04724) in contrasting DEGs for astrocytes. In astrocytes, the same contrasting pattern is also observed for other biological processes pertinent to the regulation and transmission of synaptic signaling, such as "Modulation of chemical synaptic transmission" (GO:0050804) and "Regulation of trans-synaptic signaling" (GO:0099177), as well as for molecular functions essential for synaptic activity, including "Calmodulin binding" (GO:0005516), "Monoatomic ion gated channel activity" (GO:0022839), and "Gated channel activity" (GO:0022836). Most of these pathways exhibit increased gene expression activity in PD while showing decreased activity in AD, potentially reflecting differential pathologic mechanisms underlying these neurodegenerative disorders.

#### Lipid Metabolism Dysregulation

The contrasting DEGs in oligodendrocytes display significant overrepresentation in biological processes associated with cholesterol metabolism (GO:0008203) and sterol metabolism (GO:0016125). Additionally, this alteration pattern is also observed in the KEGG pathways "Steroid biosynthesis" (KEGG, hsa00100) and "Protein processing in endoplasmic reticulum" (KEGG, hsa04141). This matches with the fact that the endoplasmic reticulum (ER) is critically involved in the synthesis of nearly all lipids, including cholesterol and phospholipids, which are essential for maintaining cellular membrane integrity and function. Furthermore, the results are in line with the previously documented dysregulation of lipid metabolism in multiple neurodegenerative disorders [52] indicating its broad relevance in these conditions. All pathways associated with lipid metabolism alterations display a decreased global expression activity in PD and an increased activity in AD, which could indicate distinct molecular and cellular mechanisms driving pathology in these disorders. In PD, the decreased activity may reflect neuronal loss and mitochondrial dysfunction leading to impaired lipid synthesis and processing capabilities. Conversely, in AD, the increased activity could be a compensatory response to amyloid-beta plaque accumulation, which disrupts membrane integrity and stimulates the need for enhanced lipid synthesis and repair processes [53].

#### Inflammation and Immune Response

Among the contrasting DEGs in microglial cells, we observed a significant enrichment of biological processes associated with immune responses, including "Myeloid cell differentiation" (GO:0030099), "Regulation of hemopoiesis" (GO:1903706), and "Regulation of leukocyte differentiation" (GO:1902105). Additionally, pathways related to inflammation, such as the "JAK-STAT signaling pathway" (KEGG, hsa04630) and the "PI3K-Akt signaling pathway" (KEGG, hsa04151), are also represented in this category. In both AD and PD, neuroinflammation and aberrant immune responses have been implicated in disease progression and pathology [54].

#### Cell Adhesion

The most significant pathways enriched in shared DEGs in astrocytes are associated with the processes "cell-substrate adhesion" (GO:0031589) and "cell-matrix adhesion" (GO:0007160). There is growing evidence that cell adhesion molecules (CAMs) play important roles in neurological disorders, influencing cell plasticity, neuroinflammation, vascular changes, and amyloid-beta (Aβ) metabolism [55–57]. In addition, alterations in CAM levels have been associated with AD in numerous studies by genetic association studies [58–61].

#### Ion Channel Activity Dysfunction

Across different cell types, we observed significant alterations in ion channel activity related processes. Specifically, the gene sets for "monoatomic ion gated channel activity" (GO:0022839) and "gated channel activity" (GO:0022836) display an overrepresentation of contrasting DEGs in astrocytes, and the processes "monoatomic ion channel activity" (GO:0005216) and "ATP hydrolysis activity" (GO:0016887) are enriched in shared DEGs in oligodendrocytes. Previous studies indicate that ion channel dysfunction in astrocytes is strongly associated with oxidative stress, neuroinflammation, and changes in pathological proteins associated with neurological disorders [62].

### Network Analysis

**Fig. 4:**
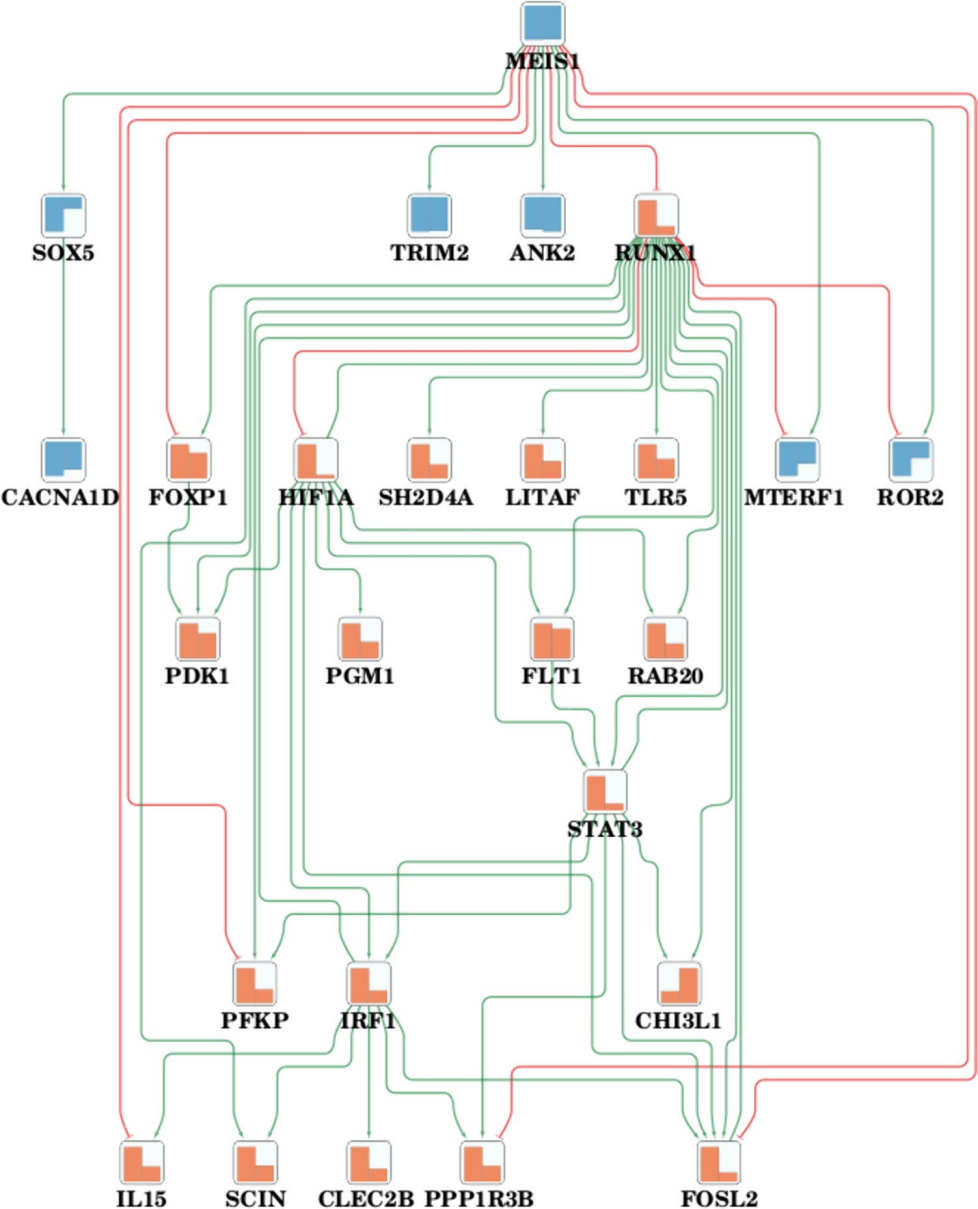
Visualization of the gene regulatory network for the top DEGs with shared patterns between AD and PD identified in microglial cells. Activating interactions are highlighted in green, inhibiting interactions in red. The colored bar plots in the nodes represent the condition-specific gene expression changes, left in PD and right in AD; increases are shown in orange and decreases in blue

To better understand gene regulatory mechanisms interlinking the identified DEGs and to determine important upstream regulators controlling these genes, we performed a gene regulatory network (GRN) analysis for the shared and contrasting DEGs (see Methods). This analysis was applied to astrocytes, oligodendrocytes, microglial cells, and excitatory neurons as the cell types with the largest number of DEGs. We built GRNs for each cell type, using the contrasting and shared DEGs. Table 4 shows each GRN top-ranked candidate regulator genes (see Methods). These candidate genes are also called perturbagens, and their activity modulation has the potential to reverse downstream pathologic gene expression changes in this network for both AD and PD (see Methods). We also identified multiple hub genes reported in Table 4 with a high network centrality (measured by the degree score) using a network topological analysis. All identified perturbagens and hub genes are listed with a brief description in Supplementary Tab 3. In addition, we searched for perturbagens overlapping across multiple cell types, to identify key regulators shared between distinct cell types. Among the perturbagens and hub genes shown in Table 4, the gene *HIF1A* stands out as a top-ranked perturbagen in the network for shared DEGs in microglial cells. *HIF1A* has a perturbation score of 4, representing the number of downstream targets DEGs whose expression can be reversed by modulating *HIF1A* activity. Moreover, *HIF1A* also displays high connectivity in multiple regulatory networks, including the network for shared DEGs in microglial cells (degree = 17, see Fig. 4) and the network for contrasting DEGs in astrocytes (degree = 23), further corroborating the relevance of *HIF1A* as a key regulator. *HIF1A* encodes a central transcriptional regulator in the HIF-1 signaling pathway, which is responsible for cellular and tissue adaptation to hypoxia. *HIF1A* also regulates genes involved in several other pathways with potential relevance in neurodegenerative disorders, such as apoptotic processes, iron and glucose metabolism, cell survival, and proliferation [63].

**Table 4:** Overview of the top perturbagens and top hub genes in networks derived from DEGs with shared or contrasting alteration patterns between AD and PD. These networks were built for 5 different cell types: astrocytes, oligodendrocytes, microglia cells, and excitatory neurons (degree: represents the total number of connections for a node; score: represents the number of downstream targets DEGs whose expression can be altered by modulating the activity of the top perturbagens)

Cell types	Category of DEGs	Top Perturbagens	Hub genes which are not found as top perturbator
Astrocyte	Shared (52 nodes, 49 edges)	CEBPD (score 23) CIITA (score 22)	MAF (degree 7) HSF1 (degree 17)
	Contrasting (401 nodes, 674 edges )	TFCP2L1 (score 81) RUNX1 (score 35) NRF1 (score 23)	LMO2 (degree 51) NRG1 (degree 23)
Oligodendrocyte	Shared (448 nodes, 800 edges)	JUNB (score 238) ZBTB7A (score 181) YY1 (score 121) RELA (score 52)	EZH2 (degree 246) FOS (degree 13)
	Contrasting (146 nodes, 167 edges )	CREB1 (score 100)	NFEDL2 (degree 13)
Microglia cell	Shared (46 nodes, 84 edges)	IRF1 (score 7) RUNX1 (score 6) HIF1A (score 4)	MES1 (degree 18)
	Contrasting (168 nodes, 320 edges)	MAF (score 97) CREB1 (score 97)	JUNB (degree 66) BCL6 (degree 25)
Excitatory Neuron	Shared (61 nodes, 65 edges)	ZEB1 (score 13)	BEND3 (degree 9) ZGLP1 (degree 7) FOS (degree 7)
	Contrasting (212 nodes, 431 edges)	HMGB1 (score 182) ZBTB7A (score 182) BRD4 (score 182) FGFR1 (score 182)	POU3F1 (degree 24) PURB (degree 12)

Apart from *HIF1A*, two additional top-scoring perturbagens identified include *JUNB* and *FOS.* They are both part of the AP-1 transcription factor family, which is involved in the regulation of cell proliferation and differentiation and, more specifically, in the regulation of inflammatory processes and T-cell signaling [64]. *JUNB* was identified as a key regulatory gene in the network of shared DEGs for oligodendrocytes (perturbation score: 238). Moreover, it was also identified as a highly connected node (degree = 66) in the regulatory network for contrasting DEGs in microglial cells, confirming its role as an important regulator of AD- and PD-associated DEGs across multiple cell types.

The gene *FOS* displayed high connectivity both in the regulatory networks for the shared DEGs in oligodendrocytes and in excitatory neurons. Furthermore, *FOS* was identified among the perturbagens in both networks for contrasting DEGs for astrocytes and microglial cells. In terms of known functional roles, *FOS* has been implicated both in neuronal survival pathways and in neuroprotective mechanisms by modulating cellular processes that enhance neuron resilience against stress [65].

Finally, one of the highest scoring regulators was the gene *CREB1*, the top-ranked perturbagen in both the network for contrasting DEGs in oligodendrocyte and in microglial cells (see Supplementary Fig. 2; perturbation scores: 100 for oligodendrocytes and 97 for microglial cells). *CREB1* is the key transcription factor in the CREB signaling pathway, which regulates neuronal plasticity by facilitating gene expression necessary for long-term potentiation, memory formation, and synaptic strength. Activation of CREB1 through various signaling pathways, including cAMP/PKA and others, leads to the transcription of genes that are important for the structural and functional changes in neurons associated with learning and memory processes [66].

### Cell-Cell Communication Analysis

We performed a cell-cell communication analysis to investigate shared patterns of altered communication events in AD and PD (see Methods). As a comprehensive analysis of all pairs of cell types is not feasible within the scope of the manuscript, we focus on the shared affected pathway results for astrocytes and microglial cells because they displayed profound and significant alterations and are of key interest in both diseases.

#### Astrocytes

When assessing disease-associated changes in cell-cell communication in astrocytes, we identified two biological processes that are commonly altered in both AD and PD, "response to hypoxia" (GO:0001666) and "positive regulation of miRNA transcription" (GO:1902893). Both displayed an overrepresentation in the target genes for the significant ligand-receptor (LR) pairs in astrocytes for both neurodegenerative diseases. Specifically, for this cell type we identified 36 significant LR pairs in the PD dataset and 45 LR pairs in the AD dataset. Notably, the process "response to hypoxia" contains the key hypoxia-associated regulatory gene *HIF1A*, which was already identified as significant in both the differential expression analysis and network analysis for astrocytes and microglia. Hypoxia has been increasingly recognized as a key factor in the pathogenesis of both AD and PD. It has been reported to accelerate the formation and accumulation of amyloid beta (Aβ) peptides, a hallmark of AD, through hypoxia-induced alterations in expression of the Aβ precursor protein (APP) and the secretase enzymes responsible for Aβ production [67–69]. Moreover, it has been described as increasing the expression and aggregation of alpha-synuclein, the key protein involved in the pathologic formation of Lewy bodies and whose aggregation contributes to the degeneration of dopaminergic neurons in PD [70].

#### Microglial Cells

In microglia, 26 biological processes were identified as significantly altered by cell-cell communication events (see complete list in Suppl. Tab. 4). Underlying these changes, 70 LR pairs were significant in the PD dataset, whereas the AD dataset contained 291 significant LR pairs. Among the biological processes enriched in the corresponding target genes, we identified a cluster of six processes jointly associated with inflammation/neuroinflammation, immune response, and apoptosis, that may reflect the contribution of immune dysregulation and cell death in the pathogenesis of AD and PD. They include the GO terms "microglial cell activation" (GO:0001774), "interleukin-6-mediated signaling pathway" (GO:0070102), "positive regulation of canonical NF-kappaB signal transduction" (GO:0043123), "positive regulation of superoxide anion generation" (GO:0032930), "extrinsic apoptotic signaling pathway" (GO:0097192) and "positive regulation of NF-kappaB transcription factor activity" (GO:0051092). In addition, a notable overlapping significant cellular process alteration between AD and PD was "growth hormone receptor signaling pathway via JAK-STAT" (GO:0060397). The JAK-STAT pathway is known to promote neuroinflammation in neurodegenerative diseases and has been proposed both as a target for pharmacological interventions in AD and as a potential predictive biomarker for AD [71]. This pathway was also highlighted as significant in the gene set enrichment analysis (see above), underscoring its relevance in the context of neurodegenerative pathology.

Overall, the cell-cell communication analysis identified shared affected cellular processes in AD and PD in astrocytes and microglia that align with the known molecular hallmarks of these diseases. Notably, the analysis also highlighted druggable pathways that have previously been proposed as potential therapeutic targets for AD or PD, and which may warrant further investigation for their applicability in broad-spectrum intervention strategies across multiple neurodegenerative conditions.

## Study limitations

While this study provides initial insights into the common and distinct molecular changes in the cortex of AD and PD, important limitations should be acknowledged.

Firstly, the use of *post-mortem* cortical tissue cannot fully reflect the *in vivo* conditions of the diseases. The *post-mortem* interval and the process of tissue degradation may affect the quality and integrity of RNA, potentially affecting the results of the scRNA-seq analyses. Although stringent quality control measures were applied, the potential for degradation remains a limitation.

Secondly, the available sample sizes and cell counts are still limited, especially for underrepresented cell types. We have therefore mainly focused on the analysis of the largest cell clusters. However, a larger sample size would provide more robust statistical power to detect subtle changes, also for smaller cell type clusters, and confirm the observed differences and similarities between AD and PD. In addition, the cohort demographics, including age and sex distribution, were not perfectly matched between the AD and PD groups, which could introduce confounding variables that could affect the results.

Thirdly, while the study focused on the cortex as a key affected brain region, neurodegenerative diseases such as AD and PD affect multiple brain regions. Focusing on the cortex may miss critical changes in other regions, such as the *hippocampus* in AD or the *substantia nigra* in PD, that are critical to understanding the full extent of these diseases.

Another limitation is the reliance on previously annotated ligand-receptor interactions to analyze cell-cell communication. Although these databases are valuable resources, they may not capture all relevant interactions or novel signaling pathways involved in AD and PD. The integration of multi-omic data, including proteomic and metabolomic profiles, could provide a more comprehensive understanding of cellular communication networks and disease mechanisms.

Finally, despite the growth of public annotation databases, the interpretation of differentially expressed genes and pathways is still inherently complex. Biological pathways are interconnected and changes in gene expression may result from compensatory mechanisms or secondary effects rather than primary disease drivers. Therefore, while this study identifies putative therapeutic targets and key regulatory pathways, further experimental validation and functional studies are essential to confirm these findings and their relevance to disease pathology.

## Conclusions

The results of the scRNA-seq analysis revealed numerous significant alterations across all considered cell types, enabling a detailed comparison of transcriptomic changes between AD and PD at single cell resolution in cortical tissue. Statistical and bioinformatic analysis highlighted both disease-specific and shared molecular signatures in AD and PD at multiple levels, including individual genes, pathways, and gene regulatory networks. Interestingly, in addition to the cellular processes that showed modulated activity in only one disease or a similar alteration trend in both diseases, we also identified a small set of contrasting pathway changes, i.e. coordinated alterations with an opposite direction between the diseases, that may reflect the same susceptible cellular processes affected by different disease mechanisms or at different stages of disease progression.

### Gene-Level Findings

We identified several significant DEGs that were either specific to AD or PD, shared between both diseases, or displayed changes with opposite directionality (contrasting DEGs). The analysis highlighted several key functional groups among these genes that may help to understand the shared and distinct cellular processes involved in these neurodegenerative disorders. Notably, genes involved in cell adhesion and cell-cell communication, such as the DEGs *CDH19, CAV1, ITGA2,* and *SELL,* play significant roles in maintaining neuronal connections and signaling. Disruptions in these processes are central to the progression of AD and PD as they lead to impaired neuronal connectivity and communication [28, 58]. Transcriptional regulation genes, including *JUNB, NR4A1,* and *FOSB*, are also prominently featured among the DEGs. Abnormal transcriptional regulation can lead to widespread alteration of gene expression, contributing to various pathologic features of neurodegenerative diseases. For instance, the identified significant Fos family genes are involved in neuroplasticity and cognitive functions, which are often impaired in AD and PD [30, 43, 65]. Similarly, genes related to neurotransmitter transport and signaling, such as the DEGs *SLC1A3* and *GABRA3,* are important for synaptic function. Dysfunctions in these genes are associated with hallmark features of AD and PD, resulting in impaired synaptic communication and neuronal death [33, 44]. Further, genes involved in cellular stress response and apoptosis, such as *SERPINB1* and *HSPA5,* are essential for managing cellular stress and preventing inappropriate cell death. The increased cellular stress and apoptosis observed in AD and PD support the potential relevance of these genes in neurodegeneration. Additionally, immune response and inflammation genes, including the DEGs *CTSZ* and *IL6R*, underscore the role of chronic inflammation in driving neuronal damage in both diseases [39, 72]. In summary, the gene-level findings emphasize the diverse molecular and cellular characteristics of AD and PD. The identified DEGs point to key relevant cellular processes, molecular hallmarks and regulatory mechanisms involved in the pathogenesis of these neurodegenerative conditions.

### Pathway-Level Findings

The pathway enrichment analyses revealed significant alterations in cellular processes for AD and PD, highlighting both distinct and overlapping changes. Contrasting alterations were particularly evident in synapse organization and signaling pathways, with PD showing a trend of increased expression and AD showing decreased expression. This may be due to differences in the neurotransmitter systems affected in each disease. PD is primarily characterized by deficits in dopamine-producing neurons in the *substantia nigra* midbrain region [4], leading to decreased dopamine levels in the basal ganglia. Activation of compensatory mechanisms in PD synaptic dysfunction has previously been described, which may result in increased activity of associated pathways [73]. Conversely, there is a more widespread disruption of multiple neurotransmitter systems in AD, including acetylcholine, serotonin, and norepinephrine. This has the potential to directly affect synaptic function and lead to a decrease in overall synaptic activity.

Lipid metabolism dysregulation also showed contrasting patterns, with decreased activity in PD and increased activity in AD. In PD, the decreased activity likely reflects impaired lipid synthesis and processing due to neuronal loss and mitochondrial dysfunction. In contrast, the increased activity in AD may be a compensatory response to amyloid-beta plaque accumulation, which disrupts membrane integrity and necessitates enhanced lipid synthesis and repair processes.

Finally, inflammation and immune response pathways, such as the JAK-STAT and PI3K-Akt signaling pathways, stood out as differentially altered between AD and PD, underscoring the important role of chronic inflammation in both diseases.

In summary, these analyses highlight essential cellular processes and pathways involved in the molecular landscape of AD and PD, indicating common and distinct disease mechanisms and potential therapeutic targets. Further validation in independent studies is recommended to confirm these results and explore their therapeutic potential.

### Network-Level Findings

The gene regulatory network analysis provided insights into shared and disease-specific, coordinated cellular process changes and key upstream regulatory genes that modulate them. Specifically, the network perturbation analysis identified *JUNB, FOS* and *HIF1A* as potential upstream regulators of numerous DEGs in AD and PD. These genes play central roles in transcriptional processes with previously confirmed relevance to neurodegeneration. For example, *JUNB* and *FOS* regulate inflammatory pathways that are known to display significant alterations contributing to chronic inflammation in AD and PD [30], and *HIF1A* modulates apoptotic and survival processes in response to hypoxic conditions, impacting cell death and survival mechanisms in degenerative disorders [67]. In addition, the transcription factor *CREB1* was identified as a key regulator among the contrasting DEGs for both oligodendrocytes and microglial cells, with the potential to reverse the expression changes for many DEGs in both cell types. In general, *CREB1* can alter the expression of genes related to neuronal growth, survival, and plasticity, and has been proposed as a drug target to ameliorate cognitive decline in aging and cognitive disorders [66].

Overall, given their central role in the GRNs, the identified key regulators—*JUNB, FOS, HIF1A,* and *CREB1*— may warrant further investigation as important mediators of pathologic processes and putative targets for the preclinical study of disease-modifying interventions.

### Cell-Cell Communication Analysis Findings

To improve our understanding of cell-cell communication disruptions in AD and PD, we applied a novel statistical approach to analyze and score changes in inter- and intra-cellular communication patterns, integrating single-cell data with known transcriptional regulatory network data and receptor-transcription factor associations from public databases. Focusing on astrocytes and microglial cells as key affected cell types with pronounced cellular process alterations in both diseases, we identified multiple pathways as commonly affected by changes in cell-cell communication events.

In astrocytes, we identified two significant pathways that are modulated by cell-cell communication events. Among these, the "response to hypoxia" pathway is not only in line with hypoxia-induced neuroinflammation as a previously proposed mechanism in degenerative disorders [74], but also matching with the results from the network analyses, highlighting the hypoxia-inducible factor *HIF1A* as a key regulator of downstream gene network expression changes in AD and PD (see above).

In contrast, for microglial cells, a larger number of significant pathways that are influenced by cell-cell communication events were identified. The majority of these pathways are associated with inflammatory and immune responses. Of particular note is the JAK-STAT signaling pathway, which was also identified as significant in the pathway enrichment analyses.

**Fig. 5:**
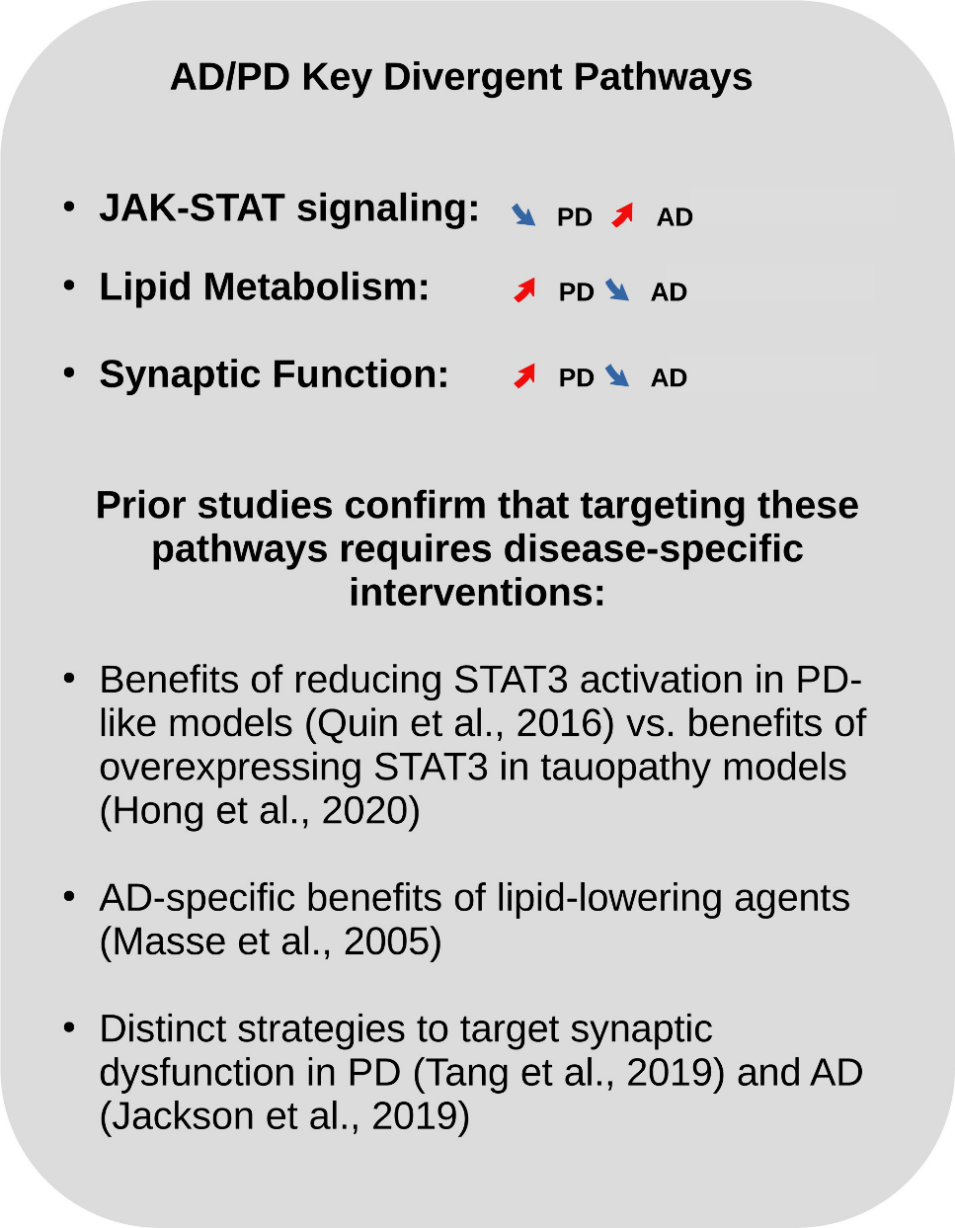
Summary of key pathways identified with divergent expression changes in AD and PD, as well as relevant findings from the biomedical literature, suggesting that disease-specific interventions are required to address the disease-specific pathologic changes in these pathways. As a key finding of this study, we identified multiple cellular pathways with significant opposing alterations between AD and PD, with supporting evidence from multiple analyses (pathway enrichment analysis, differential expression analysis for individual genes, and cell-cell communication analysis). The arrows indicate whether the associated gene expression shows an increased (↗) or decreased (↘) trend in PD (left arrow) and in AD (right arrow). The prior findings reported in the literature support both the relevance of these pathways as potential drug targets in neurodegenerative disorders, and confirm that due to distinct mechanisms in the two diseases, different drug targeting strategies may be required in AD and PD

Overall, as a key finding of our study, we have identified pathways that are significantly altered in both AD and PD and that show strongly divergent patterns in the direction of expression changes. Pronounced opposite changes were observed in particular in the JAK-STAT pathway, which mediates cytokine signaling and regulates inflammatory responses in the brain (see summary of key pathway findings across multiple analyses in Fig. 5). While previous preclinical studies have shown that JAK-STAT inhibitors can reduce inflammation and provide neuroprotection in models of neurodegenerative disease [75, 76], our results are novel in demonstrating the divergent alteration of JAK-STAT signaling in AD and PD across both pathway enrichment and cell-cell communication analyses. These findings suggest that although this pathway is implicated in both disorders, disease-specific therapeutic targeting approaches may be required to address the diverse alterations in this process. Interestingly, within the JAK-STAT pathway, reducing STAT3 activation has shown beneficial effects in a rat model of PD-like pathology [76]. Conversely, in a mouse model of tauopathy expressing the human P301L mutant tau (P301L-hTau), overexpression of STAT3 was beneficial in rescuing P301L-hTau-induced synaptic and cognitive deficits [77]. These previous results are consistent with our data, suggesting that disease-specific alterations in the JAK-STAT pathway may also require tailored therapeutic interventions specific to each disease.

Further pathways with pronounced opposing expression patterns between the diseases include lipid metabolism pathways (increased activity in AD, decreased activity in PD) and synaptic function pathways (decreased activity in AD, increased activity in PD; see Fig. 5, right size). While these pathways have previously already been proposed as potential therapeutic targets in neurodegenerative diseases [51, 52], the contrasting patterns of change between AD and PD observed in our study represent a new and unexpected discovery. Complementary mechanistic studies are required to confirm these contrasting patterns and to fully elucidate the underlying signaling pathways, but similar to the JAK-STAT pathway, the significant divergent changes identified here suggest that disease-specific strategies may be required to effectively address pathological changes in these particular processes. For example, the use of lipid-lowering agents has been proposed to be associated with slower cognitive decline in AD [78] and reduced risk of dementia [79], whereas protective effects of lipid supplementation strategies have been reported in PD [78, 80, 81]. In addition, recent studies have highlighted that targeting synaptic dysfunction in AD and PD may require different strategies due to their distinct underlying mechanisms. In AD, synaptic dysfunction is primarily associated with the accumulation of Aβ plaques and tau protein tangles, and therapeutic strategies for AD have therefore focused on targeting these proteinopathies [82, 83]. Approaches aimed at modulating neuroplasticity and synaptic maintenance are emerging as complementary to traditional therapies targeting Aβ and tau [84]. In contrast, PD is associated with synaptic dysfunction primarily due to disruption of synaptic vesicle recycling [85]. Therefore, targeting synaptic dysfunction in PD is more commonly associated with strategies that enhance synaptic vesicle recycling [86, 87].

Taken together, these findings from the literature and single cell data analysis underscore the importance of tailored therapeutic strategies to effectively target the distinct and shared molecular mechanisms in AD and PD.

## Supplementary Information

ESM 1 The supplementary materials contain visualizations of cell type marker gene expression across different cell type clusters, visualizations of gene regulatory subnetworks enriched in significant differentially expressed genes, functional annotations for identified differentially expressed genes, and shared significant pathways between AD and PD identified in the cell-cell communication analysis for astrocytes (supplementary.pdf). (PDF 561 kb)

## Data Availability

The Parkinson’s disease dataset used in this study is available in the Gene Expression Omnibus (GEO) database (ID: GSE202210). The Alzheimer’s disease dataset is available in the AWS repository on the following website: https://registry.opendata.aws/allen-sea-ad-atlas.
