# Supplementary material for: Single-Cell Cortical Transcriptomics Reveals Common and Distinct Changes in Cell-Cell Communication in Alzheimer’s and Parkinson’s Disease
Source: Mol Neurobiol. 2024 Aug 15;62(3):2655–73. doi: 10.1007/s12035-024-04419-7 (PMC11790751; doi:10.1007/s12035-024-04419-7)
Supplement: Supplementary file 1 — The supplementary materials contain visualizations of cell type marker gene expression across different cell type clusters, visualizations of gene regulatory subnetworks enriched in significant differentially expressed genes, functional annotations for identified differentially expressed genes, and shared significant pathways between AD and PD identified in the cell-cell communication analysis for astrocytes (supplementary.pdf). (PDF 561 kb) [file 12035_2024_4419_MOESM1_ESM.pdf]

Supplementary Material to *"Single-Cell Cortical Transcriptomics Reveals Common and Distinct Pathway Activity Signatures in Alzheimer's and Parkinson's Disease"*

Sophie Le Bars[1] and Enrico Glaab *[1]

[1]Luxembourg Centre for Systems Biomedicine (LCSB), University of Luxembourg, 7 avenue des Hauts Fourneaux, Esch-sur-Alzette, L-4362, Luxembourg

Supplementary Figures

1 Supplementary Fig. 1 1
2 Supplementary Fig. 2 2

Supplementary Tables

1 Supplementary Tab. 1 3
2 Supplementary Tab. 2 3
3 Supplementary Tab. 3 4
4 Supplementary Tab. 4 4

*enrico.glaab@uni.lu

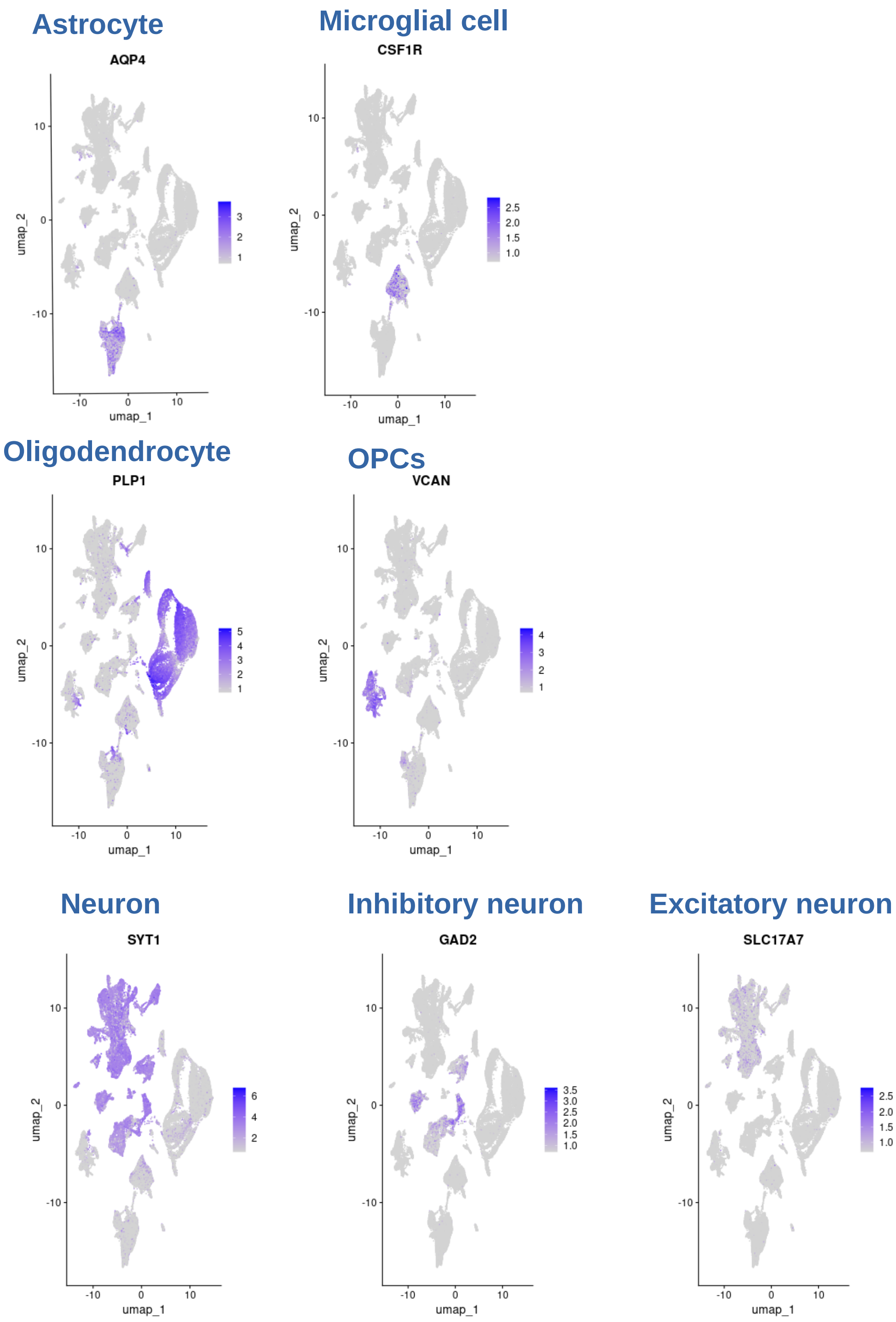

Supplementary Fig. 1: Visualization of marker gene expression across different clusters for the PD cohort. The gene markers are among the top markers for cluster differentiation and are referenced as cell type markers in the CellMarker database.

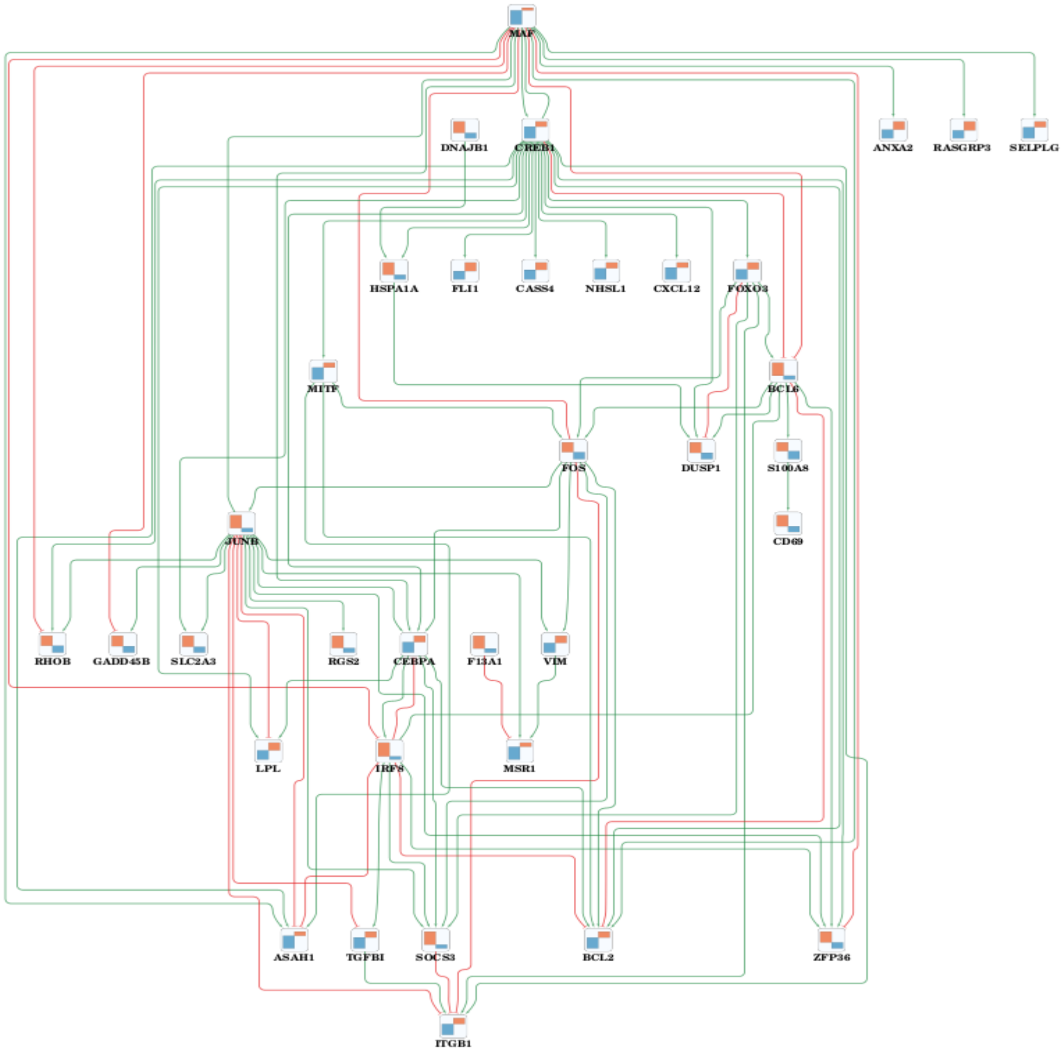

Supplementary Fig. 2: Visualization of the gene regulatory sub-network for the contrasting significant DEGs identified across the microglial cell with different change for AD and PD. Activating interactions are highlighted in green, inhibiting interactions in red. The colored bar plots in the nodes represent the condition-specific gene expression changes: left in PD and right in AD; increases are shown in red and decreases in blue.

Supplementary Tab. 1: Detailed annotations for each gene presented in Table 2 "Overview of the most significant differentially expressed genes (DEGs)".

Table is too large to be displayed.
It is hosted on a dedicated webpage (https://zenodo.org/records/10012452).

Supplementary Tab. 2: Differentially expressed genes (DEGs) annotated as neurotrophic or neuroprotective in the database NeuroProDB. Over-expressed genes are highlighted in red, underexpressed genes in blue. For the contrasting DEGs with opposite directionality in PD and AD, arrows indicate whether their expression increases (↗) or decreases (↘) in PD (left arrow) or AD (right arrow).

	Astrocyte	Oligodendrocyte	Excitatory neuron	Inhibitory Neuron	Microglial Cell
**AD-specific**	STIP1	NMNAT2<br>FGF13	CRH	–	PDGFB
**PD-specific**	–	–	–	–	–
**Shared**	VEGFA<br>GAP43	VEGFA<br>VEGFB<br>SIRT2<br>CXCR4<br>YY1<br>NTRK3<br>GRIN2B<br>PARK7<br>STIP1<br>CHL1<br>BOK	FGF1<br>PINK1<br>FGF9<br>CRLF3<br>TGFB1	VEGFA<br>VIP	STAT3
**Contrasting**	VEGFC ↘↗<br>HGF ↘↗<br>STAT3 ↘↗<br>APP ↗↘<br>MT3 ↗↘<br>GRIN2A ↗↘<br>GRIN2B ↗↘<br>ATF3 ↗↘<br>NRG1 ↗↘<br>BOK ↗↘	–	AQP4 ↗↘<br>VGF ↘↗<br>CPE ↘↗<br>STIP1 ↘↗	MT3 ↗↘	FOXO3 ↘↗

Supplementary Tab. 3: Overview of candidate genes identified in the network perturbation analysis of the shared and Contrasting DEGs in AD and PD across astrocyte, oligodendrocyte, microglial cell and excitatory neuron. Column 1 shows the gene symbols, column 2 the full gene names, and column 3 summarizes the genes' functional roles.

Table is too large to be displayed.
It is hosted on a dedicated webpage
(https://zenodo.org/records/10012452).

Supplementary Tab. 4: Overview of the common Gene Ontology pathways significantly altered by cell-cell communication events from the cell-cell communication analysis in microglial cell.

Table is too large to be displayed.
It is hosted on a dedicated webpage
(https://zenodo.org/records/10012452).
